# Role of *Sesamia nonagrioides* and *Ostrinia nubilalis* as Vectors of *Fusarium* spp. and Contribution of Corn Borer-Resistant Bt Maize to Mycotoxin Reduction

**DOI:** 10.3390/toxins13110780

**Published:** 2021-11-04

**Authors:** María Arias-Martín, Miriam Haidukowski, Gema P. Farinós, Belén Patiño

**Affiliations:** 1 Laboratory of Applied Entomology for Human and Plant Health, Centro de Investigaciones Biológicas Margarita Salas, Ramiro de Maeztu 9, 28040 Madrid, Spain; arias.maria@inia.es; 2 Institute of Sciences of Food Production, CNR, Via Amendola 122/O, 70126 Bari, Italy; miriam.haidukowski@ispa.cnr.it; 3 Department of Genetics, Physiology and Microbiology, Faculty of Biology, University Complutense of Madrid, José Antonio Novais 12, 28040 Madrid, Spain

**Keywords:** *Fusarium* spp., mycotoxins, fumonisins, Bt maize, *Sesamia nonagrioides*, *Ostrinia nubilalis*, corn borers, Spain

## Abstract

Maize expressing Cry1Ab insecticidal toxin (Bt maize) is an effective method to control *Sesamia nonagrioides* and *Ostrinia nubilalis*, the most damaging corn borers of southern Europe. In this area, maize is prone to *Fusarium* infections, which can produce mycotoxins that pose a serious risk to human and animal health, causing significant economic losses in the agrifood industry. To investigate the influence of corn borer damage on the presence of *Fusarium* species and their mycotoxins, Bt maize ears and insect-damaged ears of non-Bt maize were collected from commercial fields in three Bt maize growing areas in Spain, and differences in contamination were assessed. Additionally, larvae of both borer species were collected to evaluate their role as vectors of these molds. Non-Bt maize ears showed significantly higher presence of *F. verticillioides*, *F. proliferatum*, and *F. subglutinans* than Bt maize ears. For the first time, *Fusarium* species have been isolated from larvae of the two species. The most frequently found mycotoxins in ears were fumonisins, with non-Bt ears being significantly more contaminated than those of Bt maize. High levels of fumonisins were shown to correlate with the occurrence of corn borers in the ear and the presence of *F. verticillioides* and *F. proliferatum*.

## 1. Introduction

Maize (*Zea mays* L.) is one of the most important agricultural crops worldwide, being fundamental in the production chains of food and feed. According to the Food and Agriculture Organization of the United Nations, the global production for maize in 2019 was 1.1 billion tons, of which 132.8 million tons were produced in Europe and 4.2 million tons in Spain [1]. One of the main problems regarding maize production is mycotoxin contamination. Mycotoxins are secondary metabolites naturally produced by several filamentous fungi that grow on numerous foodstuffs such as cereals, dried fruits, nuts, and spices [2]. Mycotoxins can be produced both before and after harvest, including during storage and processing [3], and the most usual way in which they enter the food chain is through contaminated crops, mainly cereals, which are intended for food and feed. In terms of food safety, they are the most important risk associated with cereal consumption [4]. Mycotoxin contamination is not only a health problem, but also causes significant economic losses in the agrifood industry by reducing the quality of the raw materials, as well as in the livestock sector, since the consumption of products contaminated by animals of zootechnical interest leads to a decrease in reproductive rates and an increase in morbidity and mortality [5,6,7]. Even though in most cases the concentrations of mycotoxins in the feed supply chain are low enough to ensure compliance with the limits set by the European Union (EU), multi-mycotoxin contamination can increase the risk of mycotoxicosis in cattle due to additive/synergistic interactions [8,9,10].

The most relevant fungal genera affecting maize are *Fusarium* and *Aspergillus*. In Europe, *F. verticillioides, F. proliferatum, F. graminearum, F. sporotrichioides*, and *F. subglutinans* are the most frequently isolated species from maize infected by plant pathogenic fungi [11], with *F. verticillioides* as one of the main pathogens of maize, causing ear and stalk rots [12]. The main mycotoxins associated with maize during all of its production cycles and its storage are fumonisins (FUMs), trichothecenes (TCTs), zearalenone (ZEA), aflatoxins (AFs), and ochratoxin A (OTA) [4]. The first three are mainly synthesized by different species of the genus *Fusarium*, with fumonisins being the most important mycotoxins in maize cultivation due to their frequency of occurrence [13]. In addition, southern Europe has been proven to be among the regions of the world with the highest incidence of fumonisin contamination on raw feed materials and processed feeds [9].

Insect damage to the plant is one major factor that can affect the proliferation of mycotoxigenic fungi and mycotoxin production in maize. In particular, the severity of pink rot of the ear, caused by different *Fusarium* species, is closely related to the mechanical damage caused by corn borers [14]. In Spain, maize cultivation is particularly affected by two key species of corn borers, *Ostrinia nubilalis* and *Sesamia nonagrioides*. Several works have studied the relationship between mycotoxin concentration and damage produced by *O. nubilalis*, being one of the main maize pests in the USA and Europe [15,16,17]. In addition, fungi would more easily enter the plant through wounds produced by the larvae [17]. However, due to the limited distribution of *S. nonagrioides*, which is restricted to the countries of the Mediterranean area, little fieldwork has studied its involvement in maize contamination with mycotoxins [18,19,20]. A field research found that there was a good correlation between fumonisin contamination and the degree of insect damage by *S. nonagrioides* [21]. Except for this study, the few works carried out so far to clarify the hypothesis of corn borer larvae as vector of fungal infections have focused on *O. nubilalis* and *F. verticillioides* [22,23], being unknown the implication that *S. nonagrioides* could also have in the transport of different species of mycotoxigenic fungi.

Genetically modified (GM) maize varieties expressing the insecticidal toxin Cry1Ab have been suggested to affect infestation rates by *Fusarium* species, and therefore, the mycotoxin levels occurring in the commodity [19,24,25,26,27,28]. The hypothesized effect of these genetic events on mycotoxin production is assumed through the suppression of crop damage by insect pests that also may act as vectors of natural fungal infestation [22]. However, this intimate association between corn borer attack and the presence of mycotoxins is not always so clear, as demonstrated by the great variability of results obtained in studies in the USA [17,26], in Argentina [29], and in Europe [16,30]. Spain is the only country within the EU that has significantly adopted the cultivation of Bt maize expressing Cry1Ab toxin (event MON 810) for the control of these two pests, *O. nubilalis* and *S. nonagrioides*. However, few studies have been conducted on the effects of Bt maize on mycotoxin contamination and mycotoxin levels in Spain. In one of them, the infection levels of four *Fusarium* species in MON 810 hybrids were examined and compared with those of their near-isogenic non-Bt hybrids in experimental plots in two regions (Catalonia and Aragon) in 1999 and found that Bt maize had a lower content of fumonisins [31]. Conversely, a study in 2007 did not report significant differences in fumonisin concentration between Bt and non-Bt maize, probably due to the low levels of corn borer infestation that year [32,33]. Apart from these studies, no scientific field work has been published, except for some data provided in the reports prepared by the Group for the Evaluation of New Varieties of Extensive Crops in Spain (GENVCE) [34,35]. 

The main goal of this study is to determine the impact that the use of insect-resistant Bt maize (event MON 810) has in the contamination of maize ears with *Fusarium* mycobiota and in the levels of the mycotoxins they synthesize. For this purpose, we have evaluated, in three Spanish maize areas (Extremadura, Albacete and the Ebro Valley), fungal contamination by different *Fusarium* species and measured concentrations of mycotoxins (fumonisins, DON, T-2, HT-2, and ZEA) in undamaged ears from Bt maize fields and in damaged ears from naturally infested non-Bt maize fields. We have also analyzed the role of *O. nubilalis* and *S. nonagrioides* as vectors of the main *Fusarium* species. In this respect, the efficiency of Bt maize to control corn borers and therefore to prevent fungal disease and mycotoxin development in maize is discussed. 

## 2. Results

### 2.1. Occurrence of Fusarium Species in Maize Ears 

A total of 219 ears were collected in the three areas surveyed during the three years of study. Five of the eight species analyzed were detected in the samples: *F. verticillioides*, *F. proliferatum*, *F. subglutinans*, *F. graminearum*, and *F. sporotrichioides*, being 63% of the ears contaminated with at least one of them. *Fusarium verticillioides* was the most frequently detected species (in 36.7% of the ears), whereas the other four were found only sporadically in 8.3% (*F. subglutinans*), 7.3% (*F. proliferatum*), 5.5% (*F. graminearum*), and 0.9% (*F. sporotrichioides*) of the samples.

In Extremadura, the percentage of *Fusarium*-contaminated non-Bt maize ears was 100% in 2011 and 93.3% in 2012, while the contaminated ears from Bt maize fields (without corn borer damage) ranged from 35.0% in 2011 to 86.7% in 2012. The interaction between year and maize type factors was significant for the three most abundant *Fusarium* species, *F. verticillioides*, *F. proliferatum*, and *F. subglutinans*. Thus, the data for each year were analyzed separately, and it was found that in 2011 damaged non-Bt ears were significantly more contaminated with these three species than Bt maize (Table 1). In Albacete, the percentage of *Fusarium*-contaminated ears in 2011 was 73.3 and 86.7% for Bt and non-Bt maize, respectively, while in 2012 it was 25 and 55% for Bt and non-Bt maize. The results observed in the different *Fusarium* species from Albacete might suggest that, in general, there is a higher *Fusarium* contamination in non-Bt ears with respect to Bt ears. However, probably due to the variability of the results, no significant differences are observed between the two types of maize. In this area, the ears were mainly contaminated by *F. verticillioides*, with a significantly higher percentage in 2011. The next most common species was *F. proliferatum*, significantly more frequent in non-Bt maize (Table 1). In the Ebro Valley, the percentage of contaminated Bt maize ears was about 20% in 2012 and 30% in 2013, while it was 85% in non-Bt ears in both years. The species *F. proliferatum* and *F. subglutinans* were the most common, being significantly more frequent in non-Bt maize ears than in Bt ears (Table 1). 

### 2.2. Occurrence of Fusarium Species in Corn Borers

A total of 276 corn borer larvae were collected from non-Bt maize plants in the three study areas throughout the two years of sampling. Of these, 154 were *O. nubilalis* and 123 *S. nonagrioides*. Both corn borers were found in Albacete and the Ebro Valley, while in Extremadura, all larvae collected were of *O. nubilalis*. *Fusarium* species were present in 46% of larvae. The most frequent were *F. verticillioides* (in 33.3% of the larvae), *F. proliferatum*, and *F. subglutinans* (both in 23.2% of the larvae). More sporadically, some larvae were found with *F. graminearum* (12.3%), *F. sporotrichioides*, and *F. equiseti* (1.1% in both cases).

In Extremadura, *O. nubilalis* larvae were contaminated with *Fusarium* species in similar proportions (54.8 and 46.3% in 2011 and 2012, respectively) and no significant differences were found between years in larvae contaminated by the different fungal species. Both in Albacete and in the Ebro Valley, a great variability was observed in the percentages of contamination of *S. nonagrioides* and *O. nubilalis* larvae by *Fusarium* species, with no significant differences being found between the two corn borers. Albacete was the only area where *F. equiseti* was found, present in three *S. nonagrioides* larvae collected from the same field on two separate plants (Table 2).

### 2.3. Relationship between the Occurrence of Fusarium Species in Ears and Corn Borer Larvae

**Figure 1:**
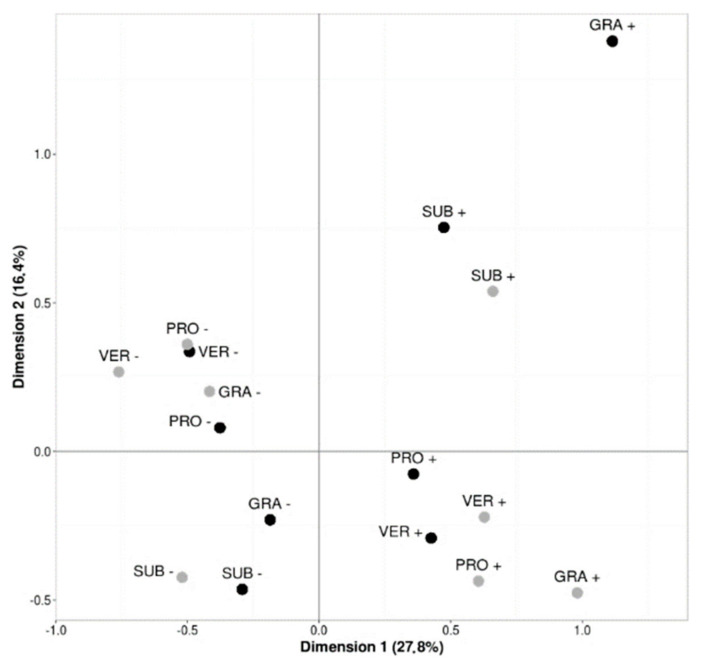
Multiple correspondence analysis (MCA) of the presence (+) or absence (−) of the toxigenic fungi found in each ear (•) and the presence (+) or absence (−) of the fungi found in some of the borer larvae (•) collected on the same plant (VER: *F. verticillioides*; PRO: *F. proliferatum*; SUB: *F. subglutinans*; GRA: *F. graminearum*).

The Multiple Correspondence Analysis (MCA) allowed relating the presence of the main fungal species isolated from corn borer larvae from non-Bt maize plants (*F. verticillioides*, *F. proliferatum*, *F. subglutinans*, and *F. graminearum*) with the presence of these fungi in the ear of the same plant (Figure 1). The first two dimensions of the MCA taken together contributed 44.2% to explain the deviation of the observed values from the expected ones. The first dimension distributed the fungal species found on the ears and on the corn borer larvae according to their presence (positive coordinates) or absence (negative coordinates). By considering the first two dimensions together, a relationship can be observed between the presence of *F. verticillioides*, *F. proliferatum*, and *F. subglutinans* in the ears and in larvae recovered from the same plant. Much clearer, as the points are farthest from the coordinate origin, is the case of *F. graminearum*, where there is no relationship between the presence of this fungus on the ear and on the larvae coming from that plant. On the other hand, the absence of the fungus on the ear is associated with the absence of the fungus on the larvae.

### 2.4. Mycotoxin Analyses

Fumonisins were the most frequently found mycotoxins in samples of Bt and non-Bt maize ears. The rest of mycotoxins analyzed (DON, T-2, HT-2 and ZEA) were only sporadically found in both types of maize (Table 3). The mycotoxin concentrations obtained for each sample analyzed are given in the Supplementary Tables S1–S6.

**Figure 2:**
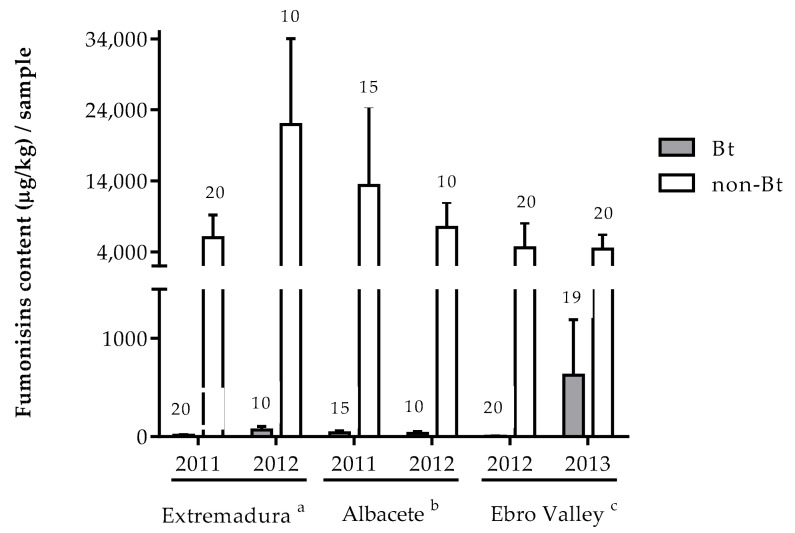
Concentration of fumonisins (mean ± S.E.) in samples of ears with borer damage (non-Bt maize) and ears without borer damage (Bt maize), collected in Extremadura, Albacete, and the Ebro Valley. The number on the bar indicates the samples analyzed.^a^ Results of the general linear model analysis in Extremadura for factor year (F = 4.29; *p* = 0.07), maize type (F = 24.07; *p* < 0.00), and interaction year × maize type (F = 0.41; *p* = 0.54); ^b^ Results of the general linear model analysis in Albacete for factor year (F = 0.01; *p* = 0.99), maize type (F = 23.39; *p* = 0.02), and interaction year × maize type (F = 0.01; *p* = 0.94); ^c^ Results of the general linear model analysis in the Ebro Valley for factor year (F = 3.46; *p* = 0.09), maize type (F = 9.33; *p* = 0.01), and interaction year × maize type (F = 0.52; *p* = 0.49).

Only for fumonisins, a sufficient number of contaminated samples was obtained to allow comparison of differences in concentration between samples from Bt and non-Bt maize fields. In all three study areas, the concentration of fumonisins was significantly higher in non-Bt maize ears than in Bt maize ears, regardless of the year of sampling. In the three regions and in all years, the average values of fumonisins found in damaged non-Bt ears exceeded the maximum limit set by the EU for unprocessed maize intended for human consumption (4000 µg/kg). The sample with the highest concentration of fumonisins found was 168,499 µg/kg, which is 42 times the maximum legislated limit and came from a non-Bt maize field in Albacete (Figure 2).

The reduction (R) in the concentration of fumonisins in Bt maize ears with respect to damaged non-Bt maize ears was over 99% in all years and in all areas except the Ebro Valley in 2013, where it was reduced by 85.6%.

## 3. Discussion

Mycotoxin contamination is one of the main problems affecting maize production due to its significant risk to human and animal health, with worldwide contamination of food crops with mycotoxins estimated at 60–80% [36]. Corn borer damage to the plant has been found to be an important driver of mycotoxigenic fungal growth and mycotoxin production [3], so the use of Cry1Ab-expressing Bt maize could lead to a reduction of both problems. Spain is the European leading country in the cultivation of Bt maize resistant to corn borers, producing 95% of the total in the European Union (EU), with 98,152 ha cultivated in 2020. Bt maize is mainly distributed in three areas of this country (the northeast (Ebro Valley), center (mainly Albacete) and southwest (Andalucia)), where this study was carried out, and which are characterized by significant attacks by *S. nonagrioides*, *O. nubilalis*, or both corn borers [37,38]. Larvae of these two lepidopteran species produce feeding wounds in kernels [39,40], so undamaged ears from Bt fields can be expected to have lower fungal growth than those from non-Bt fields in the same areas, and therefore lower levels of the mycotoxins produced by them. 

This field study demonstrates for the first time that corn borers *O. nubilalis* and *S. nonagrioides* carry mycotoxigenic fungi under Mediterranean conditions, being *F. verticillioides*, *F. proliferatum*, and *F. subglutinans* the main fungi isolated from both borers. These three fungi were also the most frequently detected in the maize ears from the three study areas, with non-Bt maize ears with borer damage showing higher contamination than corn borer-resistant Bt maize ears. The results of the ACM suggest an association between the presence of these fungi on the ears and on the larvae of the borers found in the same plants. Previous studies suggest that *O. nubilalis* can acquire fungal spores from the surface of plant debris in the soil or from an infected plant and carry them on its cuticle [23], or transfer them in its gut by feeding on a contaminated plant [22]. Our results support this, and also indicate that the same contamination pathway could be occurring in the case of *S. nonagrioides*.

Studies where mycotoxins have been identified and quantified along the various stages of the maize food chain have shown that maize grains for feed and food have the highest values of fumonisins [41]. *Fusarium verticillioides* and *F. proliferatum* have been described as the main fumonisin-producing species in Spain [42,43], with these mycotoxins the most frequently found contaminating ears in the three areas studied. The fumonisin levels were significantly higher in non-Bt corn ears than in Bt corn ears. In fact, in all three regions and in all years, average values of fumonisins exceeding the EU maximum limit for unprocessed maize for human consumption were found in the non-Bt insect-damaged ears, although it must be taken into account that in this study targeted sampling was done, i.e., selecting ears with borer damage. There could be factors unrelated to corn borer damage responsible for the differences in fumonisin levels found between Bt and non-Bt maize. However, a complementary study in which we analyzed the content of various mycotoxins in non-Bt maize ears without borer damage showed that there were no differences in the level of fumonisins between these samples and those of Bt maize collected in the same region and year (data not shown). Therefore, corn borer damage in non-Bt maize would be directly related to the high concentrations of this mycotoxin. Interestingly, the Ebro Valley is the region in Spain with the highest level of adoption of Bt maize (over 60% in the last five years) [44], and the findings of this study indicate that this is the region with lowest incidence of fumonisins and their producer fungi in both Bt and non-Bt maize. Our results are consistent with a meta-analysis of genetically engineered (GE) maize by analyzing the peer-reviewed literature [45], whose results clearly indicate that GE maize grain contains lower amounts of mycotoxins (29%), and fumonisin (31%) than its non-GE counterpart. This author also shows that the lower mycotoxin content seems to be related to the lower incidence of insect attack, since GE maize resulted in 59.6% less damaged ears compared to the corresponding isolines or near isolines. The reduction in fumonisin content that Bt corn provides by reducing insect damage has already been described in other studies in the Americas [29,46,47] and in Europe [16,19,30,31,48]. This field work demonstrates for the first time in different geographical areas of Spain, where ~95% of European Bt maize is grown, that corn borer-damaged ears have higher levels of fumonisin contamination than undamaged ears. Therefore, corn borer-resistant Bt maize expressing the Cry1Ab toxin (event MON 810), which is free of *S. nonagrioides* and *O. nubilalis* damage, would be contributing significantly to the reduction of fumonisins in maize in the areas where it is planted, which would benefit both industry and human and animal health.

The association found between *F. subglutinans* and fumonisins in maize ears could be due to the fact that this fungus is usually associated with the presence of *F. verticillioides* and *F. proliferatum*, these three species being the main causes of pink rot of the ear. The occurrence of ears contaminated with *F. graminearum* and *F. sporotrichioides* was considerably lower in both types of corn and no influence of the type of maize (Bt and non-Bt maize) on their occurrence was observed. The penetration of these species into the ear usually occurs through the silk [49] and therefore would not depend on insect damage, so that protection against borers would not prevent infection with these fungi. While in a previous study a 37% reduction of trichothecenes contamination in Bt corn has been observed [45], in our work, we have not found differences between both types of corn, having few samples contaminated with these toxins. The lower incidence of these mold species in Spain could be related to the range of optimal environmental conditions for their development [50]. In addition, it has been described that *F. graminearum* can coexist with fumonisin-producing species, since they share a host and have a much higher growth rate than them, although its development is reduced when ecophysiological conditions are favorable to *F. verticillioides* and *F. proliferatum* [51].

Different studies have suggested that the high temperatures and drought expected to occur in a climate change scenario could lead to more frequent occurrence of fumonisins in Mediterranean countries [52,53,54]. These conditions could favor the proliferation of *F. verticillioides*, closely related to the presence of borers, compared to other *Fusarium* species common in cereals, such as *F. graminearum*, which has less tolerance to heat and water stress [55,56] and, as we have proved in this work, depends less on the damage produced by corn borers to contaminate the ear. Bt maize in this new scenario could represent an effective alternative to prevent the entry of fumonisins into the food chain. Moreover, prevention is the best way to minimize fungal contamination and mycotoxin production. Therefore, in a European context, the use of Bt maize could be incorporated into good agricultural practices (GAP) [57] and in integrated pest management programs as is the case in Spain [58], by minimizing the damage caused by its main pests, which is an attractive crop option compared to conventional corn in areas of high infestation, promoting production and crop quality.

## 4. Materials and Methods

### 4.1. Collection and Processing of Maize Ears and Corn Borers

Sampling was carried out in commercial fields of different varieties of transgenic maize plants (event MON 810) expressing the toxin Cry1Ab from *Bacillus thuringiensis* (Bt maize) and of maize near-isogenic line (non-Bt maize) (Table S7).

Five plants were selected in each field, distributed throughout the plot, separated from each other and with the border at least 20 m. In the fields of Bt maize, the selection was random, since no plants showed any corn borer damage (Figure S1). Selected plants from Bt maize fields were tested for expression of Cry1Ab toxin during sampling by the Bt-Cry1Ab/1Ac ImmunoStrip^®^ test (Agdia, Elkhart, IN, USA), following the manufacturer’s instructions. In the non-Bt fields the sampling was directed, selecting plants with visible corn borer damage in the ear (Figure S1). From each plant, the ear was collected and stored in a hermetic plastic bag. In addition, in non-Bt fields the cane was checked for *O. nubilalis* and *S. nonagrioides* larvae. The insects found were stored individually in small plastic boxes (4 cm diameter × 2 cm high) with a piece of cane from the same plant in which they were found so that they would arrive alive at the laboratory. 

In the laboratory, the ears were shelled and inspected for corn borers. The insects found were stored individually in small plastic boxes (4 cm diameter × 2 cm high). The grains of each ear were dried in an oven (T 6060, Heraeus instruments, Hanou, Germany) at 65 °C for 24 h and then ground with a granite stone mill (Fidibus XL, Conasi, Spain). The flour was stored in paper bags at 4 °C. The insects collected from the cane and the ears were individually frozen at −20 °C.

Sampling was conducted in 3 to 4 different fields located in three maize growing areas of the Iberian Peninsula where Bt maize is widely adopted: the Autonomous Community of Extremadura, the province of Albacete and the Ebro Valley region. In each of them, samples were taken during two consecutive years, between 2011 and 2013 (2011 and 2012 in Extremadura and Albacete, and 2012 and 2013 in the Ebro Valley). Samples of ears were collected from 3 to 4 maize fields per area and year. These were taken just before the harvest, which took place approximately in mid-September in Extremadura, early October in Albacete, and mid-October in the Ebro Valley. The crops were maintained according to local agronomical practices, excluding the use of insecticides (Table S7).

### 4.2. Molecular Identification of Fungi Producing Mycotoxins in Flour Maize and Corn Borers. DNA Extraction and Specific PCR Detection

One gram of each pool flour maize or an individualized larva were cultured in 250 mL Erlenmeyer flasks containing 50 mL Sabouraud liquid medium (Scharlau Chemie, Barcelona, Spain) supplemented with chloramphenicol (0.5%) and incubated in darkness for 24 h for the flour and for 48 h for the insects at 28 ± 1 °C in an orbital shaker (140 rpm) (711, VDRL, Asal, Milan, Italy). Fungal mycelia were filtered through filter paper Whatman No 1 (Whatman International Ltd., Maidstone, UK) and stored at −80 °C until processing. For DNA extraction, each sample was frozen with liquid nitrogen and grinded using a mortar and a pestle.

For samples of maize flour, DNeasy Plant Mini Kit (Qiagen, Valencia, Spain) was used according to manufacturer’s instructions to obtain genomic DNA starting from 100 mg of filtered culture. All samples were analyzed in triplicate. The extraction of DNA from the mycelium obtained from the insects was carried out following the protocol described by [59], starting from approximately 100 mg of mycelium. The protocol was modified by adding 50 μL of Zimoliase 20T (1.5 mg/mL) (ICN Biomedicals, Santa Ana, CA, USA) and 50 μL of lytic enzymes from *Trichoderma harzianum* (2 mg/mL) (Sigma Aldrich, Hamburg, Germany) to the corresponding lysis buffer and then incubating for 1 h at 37 °C. DNA concentrations were determined using a NanoDrop^®^ ND-1000 spectrophotometer (Nanodrop Technologies, Wilmington, NC, USA). 

Presence of fungal DNA in all the samples were tested for suitability for PCR amplification using universal primers ITS1 and ITS2 [60] and the protocol described elsewhere [61]. Subsequently, previously described species-specific PCR protocols were used to detect the fumonisin-producing *Fusarium* species: *F. proliferatum* [45] and *F. verticillioides* [62]. PCR protocols were also applied to detect the trichothecenes producing *Fusarium* species [45], and subsequently, positive samples were evaluated for the presence of *F. graminearum*, *F. culmorum*, *F. equiseti*, *F. poae*, and *F. sporotrichioides* by the protocol described in [63]. The specific detection of *F. subglutinans* was performed using the protocols described in [64]. 

The PCR assays were performed in an Eppendorf Mastercycler Gradient (Eppendorf, Hamburg, Germany). Amplification reactions were carried out in volumes of 25 µL containing 200 ng of template DNA, 1 µL of each primer (20 µM), 2.5 µL of 10 × PCR buffer, 1 µL of MgCl2 (50 mM), 0.2 µL of dNTPs (100 mM), and 0.15 µL of Taq DNA polymerase (5 U/µL) supplied by the manufacturer (Biotools, Madrid, Spain). PCR products were detected in 1.5% agarose ethidium bromide gels in TAE 1 × buffer (Tris-acetate 40 mM and EDTA 1.0 mM). The 100 bp DNA ladder (MBI Fermentas, Vilnius, Lithuania) was used as molecular size marker.

### 4.3. Extraction, Detection, and Quantification of Mycotoxins

The quantification of mycotoxins in the ears was carried out in collaboration with different expert laboratories following different protocols. Since the detection limits for each mycotoxin were different in each protocol, the least favorable limit was chosen in order to treat the data obtained jointly (3 µg/kg for ZEA, 5 µg/kg for T-2, 10 µg/kg for HT-2, 35 µg/kg for DON, 10 µg/kg for FB_1_, and 19 µg/kg for FB_2_).

#### 4.3.1. Deoxynivalenol (DON), Zearalenone (ZEA), Toxins T-2, and HT-2 Analysis

The analyses of DON, ZEA, T-2 and HT-2 mycotoxins were performed by LC/MS/MS.

##### Protocol for Samples of 2011

The analyses were performed using the method of [65] in the Institute of Sciences of Food Production (ISPA-CNR). For each samples, 5 g of flour were extracted with 25 mL acetonitrile/water (84/16, *v*/*v*) by shaking for 60 min at 250 rpm. The extract was filtrated through filter paper (Whatman no. 4, Maidstone, VT, USA), and 5 mL were evaporated to dryness at 50 °C under a stream of air. The residues were dissolved with firs 100 µL methanol, vortexing for 1 min, then 900 µL water and vortexed again for 1 min. The Oasis^®^ HLB column was activated and conditioned prior to us as follows. The column was attached to vacuum manifold, conditioned with 2 mL methanol, and equilibrated with 2 mL methanol/water (10/90, *v*/*v*). Then, the reconstituted sample extract was passed through the column at flow rate of about one drop per second. The column was washed with 1 mL methanol/water (20/80, *v*/*v*) and dried. Afterwards, the toxins were eluted with 1 mL methanol. For the elute drying at 50 °C under stream of air, the residue was redissolved with 500 µL of methanol/water (20/80, *v*/*v*) by vortexing for 1 min and filtrated though 0.45 µm regenerated cellulose filter (Minisart^®^, Sartorius Stedim, Germany). LC/MS/MS analyses were performed on QTrap MS/MS system from Applied Biosystems (Foster City, CA, USA) equipped with an electrospray ionization interface and 1100 series micro-LC system comprising a binary pump and micro auto sample from Agilent Technologies (Waldbronn, Germany). The analytical column was Kinetex C18 (100 mm × 2.10 mm, 2.6 µm particle size) (Phenomenex, Torrance, CA, USA). The column was preceded by a KrudKatcher Ultra in-line filter (0.5 µm × 4mm, Phenomenex, Torrance, CA, USA). Detection limit was 3 µg/kg for ZEA, 5 µg/kg for T-2, 10 µg/kg for HT-2, and 35 µg/kg for DON.

##### Protocol for Samples of 2012 and 2013

The analyses were performed using the method of [66] in the Laboratorio Arbitral Agroalimentario (MAGRAMA). For each samples, 12.5 g of flour were extracted with 100 mL acetonitrile/water (80/20, *v*/*v*) by shaking for 30 min at 250 rpm. The extract was filtrated through filter paper (Whatman no. 4, Maidstone, USA). A total of 4 mL of the filtrate was added 25 μL of internal standard of Zearalanone (ZAN) 1 μg/mL acetonitrile/water (80:20 *v*/*v*) (Romer Labs, Butzbach, Austria). The extract was applied to solid phase extraction column (Bond Elut-Mycotoxin, Agilent Technologies, Waldbronn, Germany) at flow rate of about one drop per second. The extract was evaporated in a speed vac (Savant SC100, Thermo Fisher Scientific, Linz, Austria) at 50 °C, and the residues was dissolved with firs 100 µL methanol, vortexing for 1 min, and then 1900 µL de solution A/methanol (1/1, *v*/*v*), being solution A water with 0.1% acetic acid and ammonium acetate 5 mM, and filtrated though 0.22 µm regenerated cellulose filter (Minisart^®^, Sartorius Stedim, Göttingen, Germany). LC/MS/MS analyses were performed on 325 LC/MS (Varian Inc., Palo Alto, CA, USA) equipped with an electrospray ionization interface and a HPLC system comprising a binary pump 212 LC and micro auto sample 460 LC from Agilent Technologies (Waldbronn, Germany). The analytical column was Luna C18 (150 mm × 2 mm, 3 µm particle size, Phenomenex, Torrance, CA, USA). The column was preceded by a for in-line filter (4 mm × 2 mm, Phenomenex, Torrance, CA, USA). Detection limit was 3 µg/kg for ZEA, 5 µg/kg for T-2 and HT-2, and 5 µg/kg for DON.

#### 4.3.2. Fumonisins (B_1_ and B_2_) Analyses

The fumonisin analysis was performed by HPLC-FLD, previously derivatized with o-phthaldialdehyde (OPA) solution to form the fluorescent derivatives of the fumonisins and were detected by fluorescence.

##### Protocol for Samples of 2011

Fumonisins were measured by using the method for the extraction of AOAC Official Method 995.15 and the quantification by LC-FLD is described in [67] in ISPA-CNR. For each sample, 12.5 g of our sample were extracted with 50 mL methanol/water (75/25, *v*/*v*) by shaking for 60 min at 250 rpm. The extract was filtrated through filter paper (Whatman no. 4, Maidstone, VT, USA) and the pH of the extract was measured and adjusted to 5.8–6.5. Ten milliliters of filtrate were applied to strong anion-exchange (500 mg SAX, Varian Inc., Palo Alto, CA, USA) cartridge previously conditioned by successive washing with 5 mL methanol and 5 mL methanol/water (75/25, *v*/*v*) followed by 8 mL of methanol/water (75/25, *v*/*v*) and 3 mL of methanol. Fumonisins were eluted with 10 mL of 1% acetic acid in methanol. The elutes were evaporated to dryness under a stream of nitrogen at 50 °C. The residues were dissolved with 500 µL of acetronirile/water (30/70, *v*/*v*). The HPLC apparatus consisted of a ProStar system (Varian Inc., Palo Alto, CA, USA) equipped with a fluorometric detector set at wavelengths, ex = 335 nm, em = 440 nm. The column was a SymmetryShield C18 (150 mm × 4.6 mm, 5 µm particle size) with a guard column inlet filter (0.5 µm × 3 mm diameter, Rheodyne Inc., Rohnert Park, CA, USA). Detection limit for fumonisins was 2 µg/kg.

##### Protocol for Samples of 2012 and 2013

Fumonisins were analyzed using the method described in [68] in the Food Technology Department (UdL). The fumonisins were extracted with a mix of water/methanol/acetronirile (50/25/25, *v*/*v*/*v*). The filtrated extract was purified with a immunoaffinity column (Fumoniprep, R-Biopharm Rhône Ltd., Glasgow, UK). Fumonisins were eluted with water/methanol (50/50, *v*/*v*). The quantification was done with a Waters HPLC-system (Waters 2695, Waters Corporation, Milford, DE, USA), equipped with a fluorometric detector set at wavelengths, ex = 335 nm, em = 440 nm (Waters Multi λ Fluorescence Detector 2475). The column was a Spherisorb^®^ OSD2 C18 (150 mm × 4.6 mm, 5 μm particle size, Waters Corporation, Milford, DE, USA) with a guard column inlet filter Spherisorb^®^ S5OSD2 (100 mm × 4.6 mm, Waters Corporation, Milford, DE, USA). Detection limit was 10 µg/kg for FB_1_ and 19 µg/kg for FB_2_.

### 4.4. Statistical Analysis

Differences in the presence of fungal species of the genus *Fusarium* in ears of Bt maize (without corn borer damage) and non-Bt maize (with corn borer damage) were analyzed by a General Linear Model (GLM) with fixed and random factors. As the samples collected from the same field were subjected to the same agronomic practices and weather conditions, a percentage of contamination was calculated for each fungal species by sampling date and field, which was transformed with arcsine √x to standardize it. The fixed factors that were analyzed were year (2011, 2012, and 2013) and maize type (Bt and non-Bt), and their interactions, entering as a random factor the field nested within the year, since the same fields were not sampled in all years. Differences in the concentration of fumonisins (FB_1_ + FB_2_) between ears from Bt and non-Bt maize fields were analyzed with the same GLM model, after data transformation with Ln (x + 1) for normalization. The reduction (R) of fumonisin concentration in Bt maize ears with respect to damaged non-Bt maize ears was calculated by the following formula:

R (%) = ((Fumonisin concentration in non-Bt maize − Fumonisin concentration in Bt maize)/Fumonisin concentration in non-Bt maize) × 100, where fumonisin concentration is the average concentration per year and per area.

Finally, the same model was used to analyze the differences in the presence of mycotoxigenic fungi between *O. nubilalis* and *S. nonagrioides* larvae collected from non-Bt maize plants. For this, a percentage of contamination was calculated for each fungus species per field and corn borer species, and this value was transformed with the arcsine √x. In this case, the fixed factors analyzed were the year (2011, 2012, or 2013) and the corn borer species (*O. nubilalis* and *S. nonagrioides*), and their interaction, including as a random factor the field nested within the year. In all cases, when a significant interaction was found between the factor year and the factor maize type or corn borer species, the differences in these two variables were analyzed in each year separately. All analyses were performed with IBM SPSS Statistics v.25 (Armonk, NY, USA) [69] using a significance level of *p* < 0.05.

The relationship between the presence of the most abundant *Fusarium* species isolated from corn borer larvae (*F. verticillioides*, *F. proliferatum*, *F. subglutinans*, and *F. graminearum*) and their presence in the ears of the same plant where larvae were collected was analyzed by a Multiple Correspondence Analysis (MCA), performed with the SAS software (Version 9.4).

## Figures and Tables

**Table 1:** Ears without corn borer damage (Bt maize) and with corn borer damage (non-Bt maize) contaminated by *Fusarium* species and results of the GLM analysis.

Maize Ears Contaminated by Field (% Mean ± S.E.) ^a^								GLM Results		
Area	*Fusarium* Species	2011		2012		2013		Year	Maize Type	Year × Maize Type
		Bt	Non-Bt	Bt	Non-Bt	Bt	Non-Bt	F (*p*)	F (*p*)	F (*p*)
								(g.l. = 1)	(g.l. = 1)	(g.l. = 1)
Extremadura	*F. verticillioides*	35.0 ± 5.0	90.0 ± 5.8	86.7 ± 13.3	93.3 ± 6.7			#	#	8.96 (0.03) *
	*F. proliferatum*	5.0 ± 5.0	70.0 ± 12.9	26.7 ± 17.6	13.3 ± 13.3			#	#	13.49 (0.02) *
	*F. subglutinans*	0	80.0 ± 20.0	0	0			#	#	15.61 (0.01) *
	*F. graminearum*	0	15.0 ± 9.6	0	20.0 ± 11.6			0.13 (0.73)	6.43 (0.05)	0.13 (0.73)
	*F. sporotrichioides*	5.0 ± 5.0	10.0 ± 10.0	0	13.3 ± 13.3			0.04 (0.84)	0.7 (0.44)	0.26 (0.63)
Albacete	*F. verticillioides*	60.0 ± 23.1	86.7 ± 13.3	20.0 ± 8.2	55.00 ± 9.57			37.51 (<0.00) *	2.37 (0.18)	0.02 (0.90)
	*F. proliferatum*	0	80.0 ± 11.6	5.0 ± 5.0	50.00 ± 12.91			2.11 (0.21)	31.76 (<0.00) *	2.47 (0.18)
	*F. subglutinans*	33.3 ± 24.0	60.0 ± 23.0	5.0 ± 5.0	33.33 ± 9.57			3.7 (0.11)	4.44 (0.09)	0.89 (0.39)
	*F. graminearum*	6.7 ± 6.7	40.0 ± 23.0	0	0			22.66 (0.01) *	1.27 (0.31)	1.27 (0.31)
	*F. sporotrichioides*	0	26.7 ± 26.7	0	0			1.43 (0.29)	1.43 (0.29)	1.43 (0.29)
Ebro Valley	*F. verticillioides*			5.0 ± 5.0	15.0 ± 5.0	31.3 ± 12.7	25.0 ± 9.6	1.86 (0.22)	0.83 (0.40)	2.63 (0.16)
	*F. proliferatum*			10.0 ± 5.8	65.0 ± 15.0	0	50.0 ± 17.3	1.35 (0.29)	19.00 (<0.00) *	0.14 (0.72)
	*F. subglutinans*			10.0 ± 5.8	70.0 ± 12.9	5.0 ± 5.0	25.0 ± 18.9	4.15 (0.09)	9.9 (0.02) *	2.48 (0.17)
	*F. graminearum*			20.0 ± 14.1	20.0 ± 14.1	5.0 ± 5.0	15.0 ± 9.6	0.71 (0.43)	0.18 (0.69)	0.18 (0.69)
	*F. sporotrichioides*			0	10.0 ± 5.8	0	0	3.00 (0.13)	3.00 (0.13)	3.00 (0.13)

^a^ calculated with the percentages of contamination obtained from analyzing 5 ears of each field and maize type. The number of fields were 4 (Extremadura 2011, Albacete 2012 and Ebro Valley 2012 and 2013) and 3 (Extremadura 2012 and Albacete 2011). # results of the statistical analysis for the factors year and maize type have been omitted in the table when their interaction was significant. In this case, data were reanalyzed separating the data of the 3 years, this being the case of *F. verticillioides* (F = 24.01; *p* < 0.00 for 2011 and F = 1.00; *p* = 0.42 for 2012), *F. proliferatum* (F = 18.05; *p* = 0.01 for 2011 and F = 2.73; *p* = 0.24 for 2012), and *F. subglutinans* (F = 21.86; *p* = 0.02 for 2011) in Extremadura. * significant differences (*p* < 0.05).

**Table 2:** Larvae of corn borers (*O. nubilalis* and *S. nonagrioides*) contaminated by *Fusarium* species and results of the GLM analysis.

Corn Borers Contaminated by Field (% mean ± S.E.) ^a^								GLM Results		
Area	*Fusarium* Species	2011		2012		2013		Year	Corn Borer Species	Year x Corn Borer Species
		*O. nubilalis*	*S. nonagrioides*	*O. nubilalis*	*S. nonagrioides*	*O. nubilalis*	*S. nonagrioides*	F (*p*)	F (*p*)	F (*p*)
								(g.l. = 1)	(g.l. = 1)	(g.l. = 1)
Extremadura	*F. verticillioides*	50.1 ± 5.7		41.1 ± 4.8				1.46 (0.29)		
	*F. proliferatum*	12.5 ± 6.6		38.1 ± 12.3				3.38 (0.14)		
	*F. subglutinans*	16.2 ± 9.6		20.7 ± 15.1				0.06 (0.82)		
	*F. graminearum*	25.1 ± 7.0		25.7 ± 12.6				0.002 (0.97)		
	*F. sporotrichioides ^b^*	6.3 ± 3.3		0				-		
	*F. equiseti*	0		0				-		
Albacete	*F. verticillioides*	66.7 ± 22.0	15.8	24.8 ± 12.7	14.7 ± 11.1			0.82 (0.42)	5.07 (0.39)	1.34 (0.55)
	*F. proliferatum*	16.7 ± 16.7	2.6	55.7 ± 26.0	25.9 ± 15.8			1.2 (0.32)	0.87 (0.42)	0.45 (0.55)
	*F. subglutinans*	50.0 ± 14.4	21.1	3.3 ± 3.3	3.0 ± 3.0			11.33 (0.02) *	1.99 (0.40)	0.17 (0.75)
	*F. graminearum*	33.3 ± 16.7	10.5	0	0			1.22 (0.32)	11.84 (0.08)	11.84 (0.08)
	*F. sporotrichioides*	0	0	0	0			-	-	-
	*F. equiseti ^b^*	0	7.9	0	0			-	-	-
Ebro Valley	*F. verticillioides*			25.0 ± 14.4	61.3 ± 15.1	25.2 ± 16.2	23.8 ± 7.8	1.88 (0.20)	1.64 (0.23)	1.91 (0.19)
	*F. proliferatum*			30.0 ± 12.2	64.4 ± 14.6	14.1 ± 7.1	1.4 ± 1.4	13.52 (<0.00) *	1.03 (0.33)	4.82 (0.05)
	*F. subglutinans*			33.6 ± 22.5	69.2 ± 20.3	13.3 ± 13.3	27.7 ± 9.7	2.11 (0.19)	3.12 (0.13)	0.63 (0.46)
	*F. graminearum*			7.1 ± 7.1	3.6 ± 3.6	24.1 ± 14.5	1.4 ± 1.4	0.91 (0.37)	4.84 (0.07)	2.59 (0.16)
	*F. sporotrichioides ^b^*			1.8 ± 1.8	0	0	0	-	-	-
	*F. equiseti*			0	0	0	0	-	-	-

^a^ calculated with the percentages of contamination obtained per field and corn borer species. The number of fields were in Extremadura 4 in 2011 and 3 in 2012; in Albacete 3 for *O. nubilalis* in 2011 and 2012 and *S. nonagrioides* in 2012 and 1 for *S. nonagrioides* in 2011; and in the Ebro Valley 3 for *O. nubilalis* in 2013 and 4 for *O. nubilalis* in 2012 and *S. nonagrioides* in 2012 and 2013. ^b^ cannot be analyzed due to lack of data. * significant differences (*p* < 0.05).

**Table 3:** Ears without corn borer damage (Bt maize) and with corn borer damage (non-Bt maize) contaminated by mycotoxins (fumonisins, DON, T-2, HT-2, and ZEA).

Ears Contaminated (%) ^a^												
	Extremadura				Albacete				Ebro Valley			
	2011		2012		2011		2012		2012		2013	
	Bt	non-Bt	Bt	non-Bt	Bt	non-Bt	Bt	non-Bt	Bt	non-Bt	Bt	non-Bt
Fumonisinas (FB_1_ + FB_2_)	50.0	85.0	30.0	100.0	46.7	73.3	30.0	100.0	10.0	50.0	31.6	70.0
DON	0	5.0	0	0	0	20.0	0	0	20.0	20.0	5.3	20.0
T-2	10.0	5.0	0	0	6.7	0	0	0	0	0	0	0
HT-2	15.0	10.0	0	0	0	13.3	0	0	0	0	0	0
ZEA	0	0	0	0	0	6.7	0	5.5	0	0	0	0

^a^ The number of samples for each maize type were 10 (2012 Extremadura, 2012 Albacete), 15 (2011 Albacete), 19 (2013 Ebro Valley (Bt maize)), and 20 (2011 Extremadura, 2012 Ebro Valley, 2013 Ebro Valley (non-Bt maize)).

## Data Availability

Not applicable.

## Supplementary Materials

The following are available online at https://www.mdpi.com/article/10.3390/toxins13110780/s1, Table S1: Mycotoxin concentration (µg/kg) of samples of corn borer-damaged non-Bt maize ears and undamaged Bt maize ears collected in Extremadura in 2011; Table S2: Mycotoxin concentration (µg/kg) of samples of corn borer-damaged non-Bt maize ears and undamaged Bt maize ears collected in Albacete in 2011; Table S3: Mycotoxin concentration (µg/kg) of samples of corn borer-damaged non-Bt maize ears and undamaged Bt maize ears collected in Extremadura in 2012; Table S4. Mycotoxin concentration (µg/kg) of samples of corn borer-damaged non-Bt maize ears and undamaged Bt maize ears collected in Albacete in 2012; Table S5. Mycotoxin concentration (µg/kg) of samples of corn borer-damaged non-Bt maize ears and undamaged Bt maize ears collected in Ebro Valley in 2012; Table S6. Mycotoxin concentration (µg/kg) of samples of corn borer-damaged non-Bt maize ears and undamaged Bt maize ears collected in Ebro Valley in 2013; Table S7. Characteristics of maize hybrids evaluated for contamination by *Fusarium* species and mycotoxin contamination; Figure S1: Corn borer-damaged maize ears collected in non-Bt maize fields (a) and undamaged ear from a Bt maize field (b).
