# Supplementary material for: Role of Sesamia nonagrioides and Ostrinia nubilalis as Vectors of Fusarium spp. and Contribution of Corn Borer-Resistant Bt Maize to Mycotoxin Reduction
Source: Toxins (Basel). 2021 Nov 4;13(11):780. doi: 10.3390/toxins13110780 (PMC8620457; doi:10.3390/toxins13110780)
Supplement: Supplementary file 1 [file toxins-13-00780-s001.zip › toxins-1442446-supplementary.pdf]

Supplementary Materials: Role of *Sesamia nonagrioides* and *Ostrinia nubilalis* as Vectors of *Fusarium* spp. and Contribution of Corn Borer-Resistant Bt Maize to Mycotoxin Reduction

María Arias-Martín, Miriam Haidukowski, Gema P. Farinós and Belén Patiño

Table S1. Mycotoxin concentration (μg/kg) of samples of corn borer-damaged non-Bt maize ears and undamaged Bt maize ears collected in Extremadura in 2011.

Sample [a]		Mycotoxin [b]						
		DON	T-2	HT-2	ZEA	FB_1	FB_2	$FB_1 + FB_2$
**non-Bt maize**	RL P1	n.d.	n.d.	n.d.	n.d.	**536**	**49**	**585**
	RL P2	n.d.	n.d.	n.d.	n.d.	**378**	**65**	**443**
	RL P3	n.d.	n.d.	n.d.	n.d.	**2,309**	**551**	**2,860**
	RL P4	n.d.	n.d.	n.d.	n.d.	**33,788**	**13,427**	**47,215**
	RL P5	n.d.	n.d.	n.d.	n.d.	**24**	n.d.	**24**
	DB P1	n.d.	n.d.	n.d.	n.d.	**25**	n.d.	**25**
	DB P2	n.d.	n.d.	n.d.	n.d.	**126**	**128**	**254**
	DB P3	n.d.	n.d.	n.d.	n.d.	**27**	n.d.	**27**
	DB P4	n.d.	n.d.	n.d.	n.d.	**34,043**	**9,958**	**44,001**
	DB P5	n.d.	n.d.	n.d.	n.d.	**14,281**	**7,027**	**21,308**
	MA P1	n.d.	n.d.	n.d.	n.d.	n.d.	n.d.	n.d.
	MA P2	n.d.	n.d.	n.d.	n.d.	**133**	**37**	**170**
	MA P3	n.d.	n.d.	n.d.	n.d.	**16**	n.d.	**16**
	MA P4	n.d.	n.d.	n.d.	n.d.	n.d.	n.d.	n.d.
	MA P5	n.d.	n.d.	n.d.	n.d.	n.d.	n.d.	n.d.
	MH P1	n.d.	n.d.	**33**	n.d.	**111**	**23**	**134**
	MH P2	n.d.	n.d.	n.d.	n.d.	**83**	**20**	**103**
	MH P3	**122**	n.d.	n.d.	n.d.	**2,039**	**220**	**2,259**
	MH P4	n.d.	n.d.	n.d.	n.d.	**108**	**20**	**128**
	MH P5	n.d.	**23**	**27**	n.d.	**114**	n.d.	**114**
**Bt maize**	RL P1	n.d.	n.d.	n.d.	n.d.	**43**	n.d.	**43**
	RL P2	n.d.	n.d.	n.d.	n.d.	**16**	n.d.	**16**
	RL P3	n.d.	n.d.	n.d.	n.d.	**23**	n.d.	**23**
	RL P4	n.d.	n.d.	n.d.	n.d.	n.d.	n.d.	n.d.
	RL P5	n.d.	n.d.	n.d.	n.d.	**11**	n.d.	**11**
	DB P1	n.d.	n.d.	n.d.	n.d.	n.d.	n.d.	n.d.
	DB P2	n.d.	n.d.	n.d.	n.d.	n.d.	n.d.	n.d.
	DB P3	n.d.	n.d.	n.d.	n.d.	**44**	n.d.	**44**
	DB P4	n.d.	n.d.	n.d.	n.d.	n.d.	n.d.	n.d.
	DB P5	n.d.	n.d.	n.d.	n.d.	n.d.	n.d.	n.d.
	MA P1	n.d.	**3,491**	**6,337**	n.d.	**36**	n.d.	**36**
	MA P2	n.d.	**34**	**48**	n.d.	**22**	n.d.	**22**
	MA P3	n.d.	n.d.	**25**	n.d.	n.d.	n.d.	n.d.
	MA P4	n.d.	n.d.	n.d.	n.d.	n.d.	n.d.	n.d.
	MA P5	n.d.	n.d.	n.d.	n.d.	n.d.	n.d.	n.d.
	MH P1	n.d.	n.d.	n.d.	n.d.	**13**	n.d.	**13**

MH P2	n.d.	n.d.	n.d.	n.d.	**33**	n.d.	**33**
MH P3	n.d.	n.d.	n.d.	n.d.	**50**	n.d.	**50**
MH P4	n.d.	n.d.	n.d.	n.d.	n.d.	n.d.	n.d.
MH P5	n.d.	n.d.	n.d.	n.d.	n.d.	n.d.	n.d.

a Locations: RL: Riolobos, DB: Don Benito, MA: Madrigalejo, MH: Moheda de Gata; P: sampling point.
[b] DON: deoxynivalenol; ZEA: zearalenone; FB: fumonisins.
n.d.: not detected (<LOD).

Table S2. Mycotoxin concentration (μg/kg) of samples of corn borer-damaged non-Bt maize ears and undamaged Bt maize ears collected in Albacete in 2011.

Sample [a]		Mycotoxin [b]						
		DON	T-2	HT-2	ZEA	FB_1	FB_2	$FB_1 + FB_2$
**non-Bt maize**	MO P1	n.d.	n.d.	n.d.	n.d.	n.d.	n.d.	n.d.
	MO P2	n.d.	n.d.	n.d.	n.d.	n.d.	n.d.	n.d.
	MO P3	**282**	n.d.	n.d.	n.d.	**505**	**75**	**580**
	MO P4	**1,936**	n.d.	n.d.	**63**	**130,930**	**37,569**	**168,499**
	MO P5	n.d.	n.d.	n.d.	n.d.	**123**	**47**	**170**
	BA P1	n.d.	n.d.	**11**	n.d.	**75**	n.d.	**75**
	BA P2	n.d.	n.d.	n.d.	n.d.	n.d.	n.d.	n.d.
	BA P3	n.d.	n.d.	n.d.	n.d.	**11,180**	**4,236**	**15,416**
	BA P4	n.d.	n.d.	n.d.	n.d.	**1,773**	**920**	**2,693**
	BA P5	n.d.	n.d.	n.d.	n.d.	**6,129**	**1,629**	**7,758**
	SA P1	n.d.	n.d.	n.d.	n.d.	**58**	n.d.	**58**
	SA P2	n.d.	n.d.	n.d.	n.d.	**306**	**226**	**532**
	SA P3	n.d.	n.d.	n.d.	n.d.	**2,805**	**1,387**	**4,192**
	SA P4	**540**	n.d.	n.d.	n.d.	**89**	n.d.	**89**
	SA P5	n.d.	n.d.	**10**	n.d.	n.d.	n.d.	n.d.
**Bt maize**	MO P1	n.d.	n.d.	n.d.	n.d.	n.d.	n.d.	n.d.
	MO P2	n.d.	**12**	n.d.	n.d.	**14**	n.d.	**14**
	MO P3	n.d.	n.d.	n.d.	n.d.	n.d.	n.d.	n.d.
	MO P4	n.d.	n.d.	n.d.	n.d.	**14**	n.d.	**14**
	MO P5	n.d.	n.d.	n.d.	n.d.	n.d.	n.d.	n.d.
	BA P1	n.d.	n.d.	n.d.	n.d.	**70**	**32**	**102**
	BA P2	n.d.	n.d.	n.d.	n.d.	**127**	**32**	**159**
	BA P3	n.d.	n.d.	n.d.	n.d.	**232**	**32**	**264**
	BA P4	n.d.	n.d.	n.d.	n.d.	**14**	n.d.	**14**
	BA P5	n.d.	n.d.	n.d.	n.d.	**14**	n.d.	**14**
	SA P1	n.d.	n.d.	n.d.	n.d.	n.d.	n.d.	n.d.
	SA P2	n.d.	n.d.	n.d.	n.d.	n.d.	n.d.	n.d.
	SA P3	n.d.	n.d.	n.d.	n.d.	n.d.	n.d.	n.d.
	SA P4	n.d.	n.d.	n.d.	n.d.	n.d.	n.d.	n.d.
	SA P5	n.d.	n.d.	n.d.	n.d.	n.d.	n.d.	n.d.

[a] Locations: MO: Motilleja, BA: Barrax, SA: El Salobral; P: sampling point.
[b] DON: deoxynivalenol; ZEA: zearalenone; FB: fumonisins.
n.d.: not detected (<LOD).

Table S3. Mycotoxin concentration (μg/kg) of samples of corn borer-damaged non-Bt maize ears and undamaged Bt maize ears collected in Extremadura in 2012.

Sample [a]		Mycotoxin [b]						
		DON	T-2	HT-2	ZEA	FB_1	FB_2	$FB_1 + FB_2$
non-Bt maize	VR P1	n.d.	n.d.	n.d.	n.d.	n.d.	**44**	**40**
	VR P2	n.d.	n.d.	n.d.	n.d.	**67,517**	**30,364**	**97,882**
	VR P3	n.d.	n.d.	n.d.	n.d.	**50**	**62**	**112**
	VR P4	n.d.	n.d.	n.d.	n.d.	**1,092**	**521**	**1,613**
	VR P5	n.d.	n.d.	n.d.	n.d.	**40**	n.d.	**40**
	OB P1	n.d.	n.d.	n.d.	n.d.	**19,240**	**9,087**	**28,328**
	OB P2	n.d.	n.d.	n.d.	n.d.	**31**	n.d.	**30**
	OB P3	n.d.	n.d.	n.d.	n.d.	**63,984**	**23,155**	**87,139**
	OB P4	n.d.	n.d.	n.d.	n.d.	**44**	**54**	**98**
	OB P5	n.d.	n.d.	n.d.	n.d.	**2,975**	**1,058**	**4,033**
Bt maize	VR P1	n.d.	n.d.	n.d.	n.d.	n.d.	n.d.	n.d.
	VR P2	n.d.	n.d.	n.d.	n.d.	n.d.	n.d.	n.d.
	VR P3	n.d.	n.d.	n.d.	n.d.	n.d.	n.d.	n.d.
	VR P4	n.d.	n.d.	n.d.	n.d.	**252**	**82**	**333**
	VR P5	n.d.	n.d.	n.d.	n.d.	n.d.	n.d.	n.d.
	OB P1	n.d.	n.d.	n.d.	n.d.	n.d.	n.d.	n.d.
	OB P2	n.d.	n.d.	n.d.	n.d.	n.d.	n.d.	n.d.
	OB P3	n.d.	n.d.	n.d.	n.d.	**140**	**61**	**201**
	OB P4	n.d.	n.d.	n.d.	n.d.	**127**	n.d.	**130**
	OB P5	n.d.	n.d.	n.d.	n.d.	n.d.	n.d.	n.d.

[a] Locations: VR: Villar de Rena, OB: Obando; P: sampling point.
[b] DON: deoxynivalenol; ZEA: zearalenone; FB: fumonisins.
n.d.: not detected (<LOD).

Table S4. Mycotoxin concentration (µg/kg) of samples of corn borer-damaged non-Bt maize ears and undamaged Bt maize ears collected in Albacete in 2012.

Sample [a]		Mycotoxin [b]						
		**DON**	**T-2**	**HT-2**	**ZEA**	**FB_1**	**FB_2**	**$FB_1 + FB_2$**
**non-Bt maize**	GI P1	n.d.	n.d.	n.d.	n.d.	**229**	**131**	**360**
	GI P2	n.d.	n.d.	n.d.	n.d.	**98**	**54**	**152**
	GI P3	n.d.	n.d.	n.d.	n.d.	**43**	n.d.	**40**
	GI P4	n.d.	n.d.	n.d.	n.d.	**1,166**	**337**	**1,503**
	GI P5	n.d.	n.d.	n.d.	n.d.	**10,455**	**3,453**	**13,908**
	HE P1	n.d.	n.d.	n.d.	n.d.	**2,944**	**2,407**	**5,351**
	HE P2	n.d.	n.d.	n.d.	n.d.	**12,795**	**3,328**	**16,124**
	HE P3	n.d.	n.d.	n.d.	n.d.	**124**	**56**	**180**
	HE P4	n.d.	n.d.	n.d.	**56**	**1,384**	**781**	**2,165**
	HE P5	n.d.	n.d.	n.d.	n.d.	**23,802**	**10,707**	**34,509**
**Bt maize**	GI P1	n.d.	n.d.	n.d.	n.d.	**40**	n.d.	**40**
	GI P2	n.d.	n.d.	n.d.	n.d.	n.d.	n.d.	n.d.
	GI P3	n.d.	n.d.	n.d.	n.d.	**63**	**48**	**111**
	GI P4	n.d.	n.d.	n.d.	n.d.	n.d.	n.d.	n.d.
	GI P5	n.d.	n.d.	n.d.	n.d.	**165**	n.d.	**170**
	HE P1	n.d.	n.d.	n.d.	n.d.	n.d.	n.d.	n.d.
	HE P2	n.d.	n.d.	n.d.	n.d.	n.d.	n.d.	n.d.
	HE P3	n.d.	n.d.	n.d.	n.d.	n.d.	n.d.	n.d.
	HE P4	n.d.	n.d.	n.d.	n.d.	n.d.	n.d.	n.d.
	HE P5	n.d.	n.d.	n.d.	n.d.	n.d.	n.d.	n.d.

[a] Locations: GI: La Gineta, HE: La Herrera; P: sampling point
[b] DON: deoxynivalenol; ZEA: zearalenone; FB: fumonisins
n.d.: not detected (<LOD).

Table S5. Mycotoxin concentration (μg/kg) of samples of corn borer-damaged non-Bt maize ears and undamaged Bt maize ears collected in Ebro Valley in 2012.

Sample [a]		Mycotoxin [b]						
		**DON**	**T-2**	**HT-2**	**ZEA**	**FB_1**	**FB_2**	**$FB_1 + FB_2$**
**non-Bt maize**	EJ P1	**141**	n.d.	n.d.	n.d.	**15**	n.d.	**15**
	EJ P2	**1,905**	n.d.	n.d.	n.d.	n.d.	n.d.	n.d.
	EJ P3	**9,537**	n.d.	n.d.	n.d.	n.d.	n.d.	n.d.
	EJ P4	**1,701**	n.d.	n.d.	n.d.	**1,830**	**1,320**	**3,150**
	EJ P5	n.d.	n.d.	n.d.	n.d.	**500**	**750**	**1,250**
	CA P1	n.d.	n.d.	n.d.	n.d.	n.d.	n.d.	n.d.
	CA P2	n.d.	n.d.	n.d.	n.d.	n.d.	n.d.	n.d.
	CA P3	n.d.	n.d.	n.d.	n.d.	n.d.	n.d.	n.d.
	CA P4	n.d.	n.d.	n.d.	n.d.	n.d.	n.d.	n.d.
	CA P5	n.d.	n.d.	n.d.	n.d.	n.d.	n.d.	n.d.
	UR P1	n.d.	n.d.	n.d.	n.d.	n.d.	n.d.	n.d.
	UR P2	n.d.	n.d.	n.d.	n.d.	n.d.	n.d.	n.d.
	UR P3	n.d.	n.d.	n.d.	n.d.	**990**	**140**	**1,130**
	UR P4	n.d.	n.d.	n.d.	n.d.	**4,040**	**430**	**4,470**
	UR P5	n.d.	n.d.	n.d.	n.d.	**2,830**	**600**	**3,430**
	LU P1	n.d.	n.d.	n.d.	n.d.	**510**	**110**	**620**
	LU P2	n.d.	n.d.	n.d.	n.d.	**5,110**	**1,230**	**6,340**
	LU P3	n.d.	n.d.	n.d.	n.d.	**57,890**	**12,840**	**70,730**
	LU P4	n.d.	n.d.	n.d.	n.d.	**60**	n.d.	**60**
	LU P5	n.d.	n.d.	n.d.	n.d.	n.d.	n.d.	n.d.
**Bt maize**	EJ P1	n.d.	n.d.	n.d.	n.d.	n.d.	n.d.	n.d.
	EJ P2	n.d.	n.d.	n.d.	n.d.	**17**	n.d.	**17**
	EJ P3	n.d.	n.d.	n.d.	n.d.	n.d.	n.d.	n.d.
	EJ P4	n.d.	n.d.	n.d.	n.d.	n.d.	n.d.	n.d.
	EJ P5	n.d.	n.d.	n.d.	n.d.	n.d.	n.d.	n.d.
	CA P1	**69**	n.d.	n.d.	n.d.	**50**	n.d.	**50**
	CA P2	**421**	n.d.	n.d.	n.d.	n.d.	n.d.	n.d.
	CA P3	n.d.	n.d.	n.d.	n.d.	n.d.	n.d.	n.d.
	CA P4	**860**	n.d.	n.d.	n.d.	n.d.	n.d.	n.d.
	CA P5	**44**	n.d.	n.d.	n.d.	n.d.	n.d.	n.d.
	UR P1	n.d.	n.d.	n.d.	n.d.	n.d.	n.d.	n.d.
	UR P2	n.d.	n.d.	n.d.	n.d.	n.d.	n.d.	n.d.
	UR P3	n.d.	n.d.	n.d.	n.d.	n.d.	n.d.	n.d.
	UR P4	n.d.	n.d.	n.d.	n.d.	n.d.	n.d.	n.d.
	UR P5	n.d.	n.d.	n.d.	n.d.	n.d.	n.d.	n.d.
	LU P1	n.d.	n.d.	n.d.	n.d.	n.d.	n.d.	n.d.
	LU P2	n.d.	n.d.	n.d.	n.d.	n.d.	n.d.	n.d.
	LU P3	n.d.	n.d.	n.d.	n.d.	n.d.	n.d.	n.d.
	LU P4	n.d.	n.d.	n.d.	n.d.	n.d.	n.d.	n.d.
	LU P5	n.d.	n.d.	n.d.	n.d.	n.d.	n.d.	n.d.

[a] Locations: EJ: Ejea de los Caballeros, CA: Candasnos, UR: Urrea de Jalón, LU: Lucena de Jalón; P: sampling point.
[b] DON: deoxynivalenol; ZEA: zearalenone; FB: fumonisins.
n.d.: <LOD.

Table S6. Mycotoxin concentration (μg/kg) of samples of corn borer-damaged non-Bt maize ears and undamaged Bt maize ears collected in Ebro Valley in 2013.

**Sample** [a]		**Mycotoxin** [b]						
		**DON**	**T-2**	**HT-2**	**ZEA**	**FB_1**	**FB_2**	**FB_1 + FB_2**
**non-Bt maize**	AM P1	n.d.	n.d.	n.d.	n.d.	n.d.	n.d.	n.d.
	AM P2	n.d.	n.d.	n.d.	n.d.	n.d.	n.d.	n.d.
	AM P3	n.d.	n.d.	n.d.	n.d.	**130**	**150**	**280**
	AM P4	n.d.	n.d.	n.d.	n.d.	**2,570**	**1,300**	**3,870**
	AM P5	n.d.	n.d.	n.d.	n.d.	**290**	**130**	**420**
	CA P1	n.d.	n.d.	n.d.	n.d.	n.d.	n.d.	n.d.
	CA P2	n.d.	n.d.	n.d.	**11**	**2,330**	n.d.	**2,330**
	CA P3	**64**	n.d.	n.d.	**12**	n.d.	n.d.	n.d.
	CA P4	**97**	n.d.	n.d.	**13**	**11,840**	**2,440**	**14,280**
	CA P5	n.d.	n.d.	n.d.	**12**	**140**	**30**	**170**
	RB P1	n.d.	n.d.	n.d.	n.d.	**8,550**	**2,620**	**11,170**
	RB P2	n.d.	n.d.	n.d.	n.d.	**28,290**	**11,720**	**40,010**
	RB P3	n.d.	n.d.	n.d.	n.d.	**910**	**200**	**1,110**
	RB P4	n.d.	n.d.	n.d.	n.d.	**6,520**	**2,010**	**8,530**
	RB P5	n.d.	n.d.	n.d.	**11**	**2,660**	**1,280**	**3,940**
	TA P1	n.d.	n.d.	n.d.	n.d.	**330**	**140**	**470**
	TA P2	**494**	n.d.	n.d.	**223**	**280**	**170**	**450**
	TA P3	n.d.	n.d.	n.d.	n.d.	**250**	**150**	**400**
	TA P4	**884**	n.d.	n.d.	**25**	n.d.	n.d.	n.d.
	TA P5	n.d.	n.d.	n.d.	**1**	n.d.	n.d.	n.d.
**Bt maize**	AM P1	n.d.	n.d.	n.d.	n.d.	**120**	n.d.	**120**
	AM P2	n.d.	n.d.	n.d.	n.d.	n.d.	n.d.	n.d.
	AM P3	n.d.	n.d.	n.d.	n.d.	n.d.	n.d.	n.d.
	AM P4	n.d.	n.d.	n.d.	n.d.	n.d.	n.d.	n.d.
	CA P1	n.d.	n.d.	n.d.	n.d.	n.d.	n.d.	n.d.
	CA P2	n.d.	n.d.	n.d.	n.d.	n.d.	n.d.	n.d.
	CA P3	n.d.	n.d.	n.d.	n.d.	n.d.	n.d.	n.d.
	CA P4	n.d.	n.d.	n.d.	n.d.	**20**	n.d.	**20**
	CA P5	n.d.	n.d.	n.d.	n.d.	n.d.	n.d.	n.d.
	RB P1	n.d.	n.d.	n.d.	n.d.	**6,910**	**3,880**	**10,790**
	RB P2	n.d.	n.d.	n.d.	n.d.	n.d.	n.d.	n.d.
	RB P3	n.d.	n.d.	n.d.	n.d.	n.d.	n.d.	n.d.
	RB P4	n.d.	n.d.	n.d.	n.d.	n.d.	n.d.	n.d.
	RL P5	n.d.	n.d.	n.d.	n.d.	**170**	**140**	**310**
	TA P1	n.d.	n.d.	n.d.	**13**	n.d.	n.d.	n.d.
	TA P2	**58**	n.d.	n.d.	n.d.	n.d.	n.d.	n.d.
	TA P3	n.d.	n.d.	n.d.	n.d.	**290**	n.d.	**290**
	TA P4	n.d.	n.d.	n.d.	n.d.	**340**	n.d.	**340**
	TA P5	n.d.	n.d.	n.d.	n.d.	n.d.	n.d.	n.d.

[a] Locations: AM: Almudévar, CA: Candasnos, RB: Ribaforada, TA: Tafalla; P: sampling point.
[b] DON: deoxynivalenol; ZEA: zearalenone; FB: fumonisins.
n.d.: not detected (<LOD).

Table S7. Characteristics of maize hybrids evaluated for contamination by *Fusarium* species and mycotoxin contamination.

Area	Year	Location (province) [a]	Samples dates	Maize		
				Maize type	Variety (cycle)	Sowing dates
**Extremadura**	2011	Riolobos (CC)	15-sep	non-Bt	DKC6666 (700)	15-may
				Bt	DKC6667YG (700)	
		Don Benito (BA)	15-sep	non-Bt	PR33Y74 (600)	29-mar
				Bt	PR33Y72 (600)	
		Madrigalejo (CC)	15-sep	non-Bt	PR33Y74 (600)	20-abr
				Bt	PR33Y72 (600)	
		Moheda de Gata (CC)	16-sep	non-Bt	P1758 (700)	12-may
				Bt	DKC6667YG (700)	
	2012	Navalvillar de Pela (BA)	18-sep	non-Bt	DKC6666 (700)	13-abr
				Bt	DKC6667YG (700)	
		Villar de Rena (BA)	19-sep	non-Bt	PR31D58 (700)	08-may
				Bt	PR33D48 (700)	
		Obando (BA)	19-sep	non-Bt	PR31D58 (700)	20-abr
				Bt	PR33D48 (700)	
**Albacete**	2011	Motilleja (AB)	04-oct	non-Bt	PR34N43 (500)	15-abr
				Bt	PR31N28 (700)	
		Barrax (AB)	04-oct	non-Bt	DKC6721 (700)	07-abr
				Bt	DKC6667YG (700)	
		El Salobral (AB)	05-oct	non-Bt	DKC6721 (700)	06-abr
				Bt	DKC6667YG (700)	
	2012	La Gineta (AB)	02-oct	non-Bt	DKC6450 (700)	18-abr
				Bt	DKC6451YG (700)	
		Santa Ana (AB)	02-oct	non-Bt	DKC6450 (700)	20-abr
				Bt	DKC6451YG (700)	
		La Herrera (AB)	03-oct	non-Bt	-	09-abr
				Bt	PR34N44 (600)	
		Motilleja (AB)	03-oct	non-Bt	Helen (700)	05-abr
				Bt	Helen Bt (700)	
**Ebro Valley**	2012	Ejea de los Caballeros (HU)	16-oct	non-Bt	DKC6677 (600)	13-abr
				Bt	DKC6678YG (600)	
		Candasnos (HU)	16-oct	non-Bt	DKC6717 (700)	25-abr
				Bt	DCK5277YG (400)	
		Urrea de Jalón (Z)	17-oct	non-Bt	DKC6717 (700)	09-abr
				Bt	-	
		Lucena de Jalón (Z)	17-oct	non-Bt	DKC6717 (700)	11-abr
				Bt	DKC6667YG (700)	
	2013	Almudevar (HU)	15-oct	non-Bt	DKC6450 (700)	22-abr
				Bt	DKC6451YG (700)	
		Candasnos (HU)	15-oct	non-Bt	DKC6717 (700)	15-abr
				Bt	DKC6667YG (700)	
		Ribaforada (NA)	16-oct	non-Bt	DKC6630 (700)	06-abr
				Bt	DKC6451YG (700)	
		Tafalla (NA)	16-oct	non-Bt	DKC5401 (500)	20-abr
				Bt	PR33W86 (600)	

[a] Cáceres (CC), Badajoz (BA), Albacete (AB), Zaragoza (Z), Huesca (HU) and Navarra (NA).

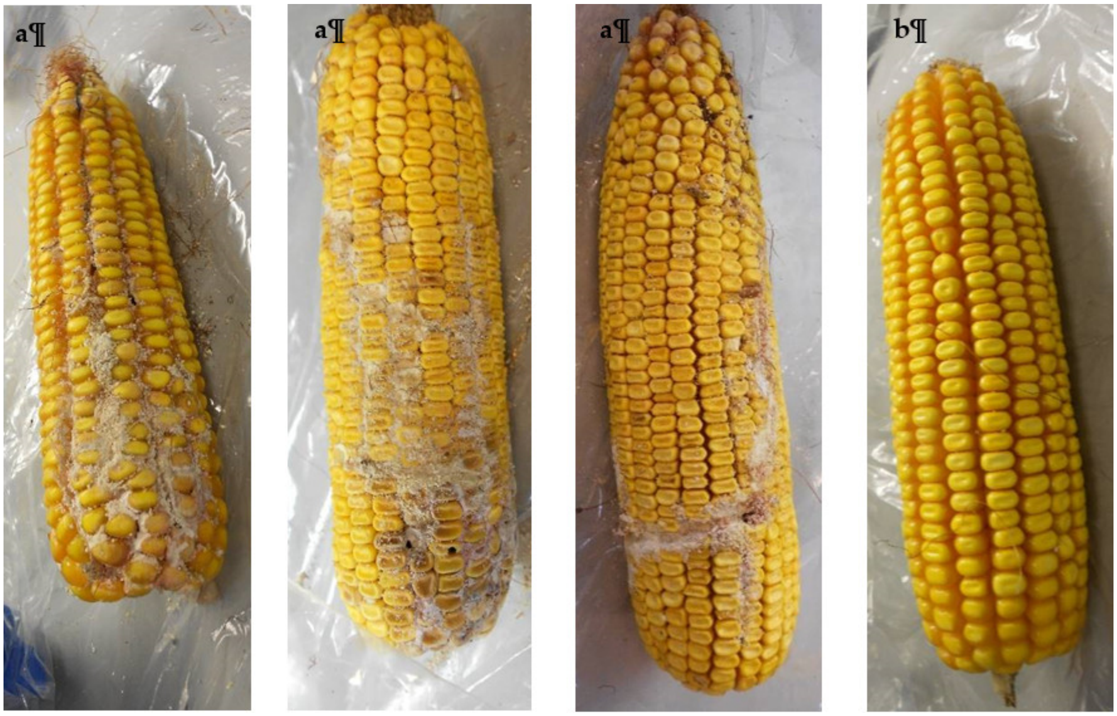

Figure S1. Corn borer-damaged maize ears collected in non-Bt maize fields (a) and undamaged ear from a Bt maize field (b).
